# Fruit-Based Beverages Contain a Wide Range of Phytochemicals and Intervention Targets Should Account for the Individual Compounds Present and Their Availability

**DOI:** 10.3390/foods9070891

**Published:** 2020-07-07

**Authors:** Charles Bestwick, Lorraine Scobbie, Lesley Milne, Gary Duncan, Louise Cantlay, Wendy Russell

**Affiliations:** University of Aberdeen Rowett Institute, Foresterhill, Aberdeen AB25 2ZD, Hong Kong; c.bestwick@abdn.ac.uk (C.B.); l.scobbie@abdn.ac.uk (L.S.); l.milne@abdn.ac.uk (L.M.); gary.duncan@abdn.ac.uk (G.D.); l.cantlay@abdn.ac.uk (L.C.)

**Keywords:** antioxidant activity, bioavailability, phenolics, sugar consumption, human studies, fruit juices, smoothies

## Abstract

Benefits from micronutrients within fruit juice and smoothies are well documented, but fewer studies research the role of phytochemicals. Well-controlled human studies are essential to evaluate their impact, particularly on glucose and lipid regulation but also gastrointestinal health. Planning these studies requires data on the potential molecular targets. Here we report a comprehensive metabolomic (LC-MS) analysis of the phytochemical composition of four commonly consumed beverages, including data on whether they are free to be absorbed early in the gastrointestinal tract or bound to other plant components. Smoothies contained a wide range of phenolics (free and bound), whereas the fruit juices contained higher amounts of fewer compounds. Orange juice was rich in bound hesperidin (1.97 ± 0.39 mg/100 mL) and hydroxycinnamic acids, likely to be delivered to the colon with the potential to have an impact on gut health. Apple juice contained free chlorogenic acid (3.11 ± 1.03 mg/100 mL), phloridzin (0.40 ± 0.03 mg/100 mL), catechin (0.090 ± 0.005 mg/100 mL), and epicatechin (0.38 ± 0.02 mg/100 mL), suggesting potential roles in glucose uptake reduction or positive effects on systemic blood flow. Redox screening established that differences in chemical composition impacted on bioactivity, highlighting the importance of availability from the matrix. This suggests that fruit-based beverage interventions should target specific mechanisms depending on the fruits from which they are comprised and in particular, the availability of the individual constituents.

## 1. Introduction

Fruits are generally considered as a heathy component of our diet and many global initiates encourage consumption of a minimum of five portions (400 g) of fruit and vegetables per day [1,2]. More recently, a meta-analysis of 95 prospective studies reported a significantly reduced relative risk for CVD, stroke, total cancer incidence and all-cause mortality with fruit and vegetables intakes in excess of 200 g daily with consumption of 800 g of fruit and vegetables per day (10 portions) considered optimal [3].

With regard to blood glucose regulation, there is substantial and increasing evidence that phytochemical-rich foods could be beneficial, but often the individual constituents responsible for this effect are poorly characterised weakening the study outcomes [4]. In light of some concerns regarding the high sugar content of some fruits and particularly fruit-based beverages, this data needs to be strengthened by more rigorous and well-designed studies, with comprehensive analysis of the active components being a key feature.

Within NHS–UK recommendations on the five-a-day consumption of fruits and vegetables, unsweetened 100% fruit juice, vegetable juice and smoothies are permitted to count as a maximum of one portion and combined total of drinks from fruit juice, vegetable juice and smoothies should not be more than 150 mL per day, reflecting the concerns and ambiguities around the consumption benefits of such beverages [5,6]. Therefore, it has become increasingly important that the role of fruit and fruit-based beverages in the diet are comprehensively analysed and that appropriate dietary interventions to explore their role in the diet are conducted. Additionally, an important aspect for consideration by the food and drink industry are differences in processing technologies, with beverages such as fruit juices and smoothies a valuable example. Moderately processed smoothies retain much more of the plant cell wall matrix, particularly insoluble wall polymers such as hemi-celluloses, lignan and protein, whereby in juice production, the insoluble pomace is removed and along with it, many of the tightly bound phytochemicals. Availability of phytochemicals depends not only on their form, but also the presence of other food components.

This paper provides a detailed description of the phytochemical characterisation of commonly consumed smoothies and fruit juices and highlights the differences in both composition and availability from the food matrix. Demonstrating the form in which phytochemicals are present is particularly important, as small molecules which are available to be absorbed early in the gastrointestinal tract can impact on postprandial events influencing systemic health. Whereas those bound to other components, commonly plant polysacchardises such as fibre and lignin can only be released and rendered bioactive with concomitant microbial transformation in the colon. These factors are essential when considering the potential health implications in the design of dietary interventions, as well as the impact of food processing procedures on both nutritional quality and benefits to health.

## 2. Methods

### 2.1. Sample Preparation

Four commonly consumed fruit beverages; orange juice (100% orange; 1G3340- 22:53; 330 mL × 4), apple juice (100% apple; 1G3341-17:56; 330 mL × 4) and mixed fruit smoothies, namely “strawberry and banana smoothie” (25% strawberry, 25% banana, plus 0.5 apple, 9 grapes, 0.5 orange, and dash of lime; 2G3333-15:47, 250 mL × 4), “mango and passion fruit smoothie” (16% mango, 3% passion fruit, plus 1 apple, 0.5 banana, small piece of peach, and dash of lime; 2G3352-15:33 250 mL × 4), were provided for analysis by Innocent ltd (London, UK). The samples were shipped by one-day courier on ice as dispatched to retailers. They were within their best before date and still within the chilled counter range. They were immediately prepared for analysis. Four bottles (1–1.32 L) of each were combined, mixed well, portioned, weighed, and frozen at −70 °C. They were freeze dried, weighed, and the lyophilised powders combined for analysis.

### 2.2. Phytochemical Extraction

The non-nutrient phytochemicals were measured by selective extraction of the free and bound (alkali and acid labile) forms. The method used was previously published [7]. Briefly, samples (approx. 0.1 g dry weight; n = 3) were suspended in HCl (0.2 mol dm^−3^; 3 cm^3^), extracted into ethyl acetate (EtOAc; 5 cm^3^) and the layers separated by centrifugation (5 min; 1800× *g*; 4 °C). The extraction was repeated three times and the EtOAc extracts combined. The organic layer was left to stand over sodium sulphate (anhydrous) for 1 h and filtered. The combined organic layers were then evaporated to dryness under reduced pressure at temperature not exceeding 40 °C and stored in a desiccator prior to analysis by LC-MS. The extract produced in this step represents the “free fraction”. The pH of the aqueous fraction was increased to pH 7 with NaOH (4 mol dm^−3^). NaOH (4 mol dm^−3^) was added to give a final concentration of 1 mol dm^−3^ and the sample stirred at room temperature for 4 h under nitrogen. The pH was reduced to pH 2 with HCl (6 mol dm^−3^) and the samples extracted into EtOAc (5 cm^3^ × 3) and processed as described above. The pH of the aqueous fraction was then increased to pH 7 with NaOH (4 mol dm^−3^). HCl (10 mol dm^−3^) was added to give a final concentration of 2 mol dm^−3^ and the sample incubated with stirring at 95 °C for 30 min. This was cooled on ice and extracted at room temperature with EtOAc (5 cm^3^ × 3) and processed again as described above. The extracts obtained after alkaline and acid hydrolysis represents the “bound fractions”. An aliquot (20 µL) of the extracts prepared above was transferred to an eppendorf. Internal standard 1 for negative mode mass spectrometry (IS1; 13C benzoic acid; 2 ng µL-1 in 75% methanol containing 0.02% acetic acid; 20 µL), internal standard 2 for positive mode mass spectrometry (IS2; 2-amino-3,4,7,8-tetramethylimidazo[4–5-f] quinoxaline; 0.5 ng µL-1 in 75% methanol containing 0.02% acetic acid; 20 µL) and acidified (HCl; 0.4 mol dm-3) methanol (40 µL) were added. The samples were mixed well, centrifuged (12,500× *g*; 5 min; 4 °C), and the supernatants analysed by LC-MS as detailed in 2.5.6.

### 2.3. Measurement of Phytochemicals by LC-MS

The LC-MS/MS analysis methods were published previously [8] Liquid chromatography separation of the metabolites was performed on an Agilent 1100 LC-MS system (Agilent Technologies, Wokingham, UK) using a Zorbax Eclipse 5µm, 150 mm × 4.6 mm C18 (Agilent Technologies, Wokingham, UK). Three gradients were used to separate the different categories of metabolites and the mobile phase solvents in each case were water containing 0.1% acetic acid (A) and acetonitrile containing 0.1% acetic acid (B). Method 1: 40%–90% B (13 min), 90% B (1 min), 90%–40% B (1 min), 40% B (9 min); method 2: 10%–55% B (45 min), 55%–80% B (15 min), 80% B (3 min), 80%–10% B (0.2 min), 10% B (4.8 min) and method 3: 50%–80% B (10 min), 80% B (2 min), 80%–50% B (1 min), 50% B (4 min). In all cases the flow rate was 300 µL min-1 with an injection volume of 5 µL. The LC eluent was directed into, without splitting, an ABI 3200 triple quadrupole mass spectrometer (Applied Biosystems, Warrington, UK) fitted with a Turbo Ion Spray™ (TIS) source. For LC methods 1 and 2, the mass spectrometer was run in negative ion mode with the following source settings: ion spray voltage -4500 V, source temperature 400 °C, gases 1 and 2 set at 15 (units) and 40 (units) respectively and the curtain gas set to 10 (units). For LC method 3, the mass spectrometer was run in positive ion mode with the following source settings; ion spray voltage 5500, source temperature 400 °C, gases 1 and 2 set at 14 (units) and 40 (units), respectively, and the curtain gas set at 10 (units). All the metabolites were quantified using multiple reaction monitoring (MRM). Standard solutions for all analytes were available from a collection held by the Rowett Institute and includes compounds purchased from commercial sources and synthesized in our laboratories. These were prepared and (10 ng µL^−1^) and pumped directly via a syringe pump. The ion transitions for each of the analytes were determined based upon their molecular ion and a strong fragment ion. For several categories of compounds, it was inevitable that their molecular ion and fragment ion would be the same, but this was overcome by their different elution times. Their voltage parameters, declustering potential, collision energy, and cell entrance/exit potentials were optimized individually for each analyte. For all of the phytochemical quantifications the standard calibrations were over a concentration interval of 2 ng μL^−1^ to 10 pg μL^−1^. The threshold used for quantification was a signal to noise ratio of 3 to 1. All the ion transitions for each of the metabolites were determined based upon their retention time, molecular ion, and a strong fragment ion; their voltage parameters; declustering potential, collision energy, and cell entrance/exit potentials were optimised individually for each metabolite and these are presented in the supplementary data (Table S1).

### 2.4. Redox Activity

The extracts prepared as described above were screened for their ability to act as redox protectants. The lyophilised extracts were standardised to a concentration of 50 mg/mL (DMSO; 50% *v*/*v*). As antioxidant activity is specific to the redox methodology, four methods were employed to capture the full spectrum of activity. These were the ferric reducing ability of plasma (FRAP), the trolox equivalent antioxidant capacity (TEAC), oxygen radical absorbance capacity (ORAC) and the hydroxyl radical antioxidant capacity (HORAC) [9]. Direct comparison of the fruit beverages was made using the Student’s t-test in Excel.

## 3. Results

The moisture content of the samples are as follows; strawberry and banana smoothie; 83.37 ± 0.15%, mango and passion fruit smoothie; 82.40 ± 0.56%, orange juice; 87.56 ± 2.33% and apple juice; 86.71 ± 0.43% and all data produced are corrected for dry matter content using these values.

The mango and passion fruit smoothie had the highest amount of total phytochemicals containing 9.36 mg/100 mL with strawberry and banana smoothie, orange juice and apple juice containing 7.69, 7.32 and 5.44 mg/100 mL respectively. The quantitative results for the phytochemical constituents in the smoothie/juice samples are detailed in Table 1, Table 2, Table 3, Table 4, Table 5, Table 6 and Table 7. Tables with the retention time and *m*/*z* of the individual compounds analysed, as well as representative chromatograms are presented as supplementary data (Table S1 and Figure S1A–D). The data is presented as phytochemicals that are free (i.e., not bound to other cell wall components) and easily solubilized into ethyl acetate and low pH and those that are bound to the plant cell wall or other components (and require liberation with alkali or acid, as described in Section 2.2). This gives an indication as to which compounds might be freely available to be absorbed early in the gastrointestinal tract, impacting postprandial events and those that could be delivered directly to the colon. Compounds delivered to the colon can also influence the gut microbiota, as well as being transformed and metabolised impacting on gut health [10].

In general, the smoothies had a wider range of phytochemicals above 0.1 mg/100 mL compared to the juices. Although orange juice had the phytochemical present at the highest concentration (Hesperidin; 3.69 ± 0.44 mg/100 mL), the strawberry and banana smoothie had the widest range of phytochemicals in this concentration range. The strawberry and banana smoothie was rich in hesperidin, ferulic acid, p-coumaic acid, and chrologenic acid. The mango and passion fruit smoothie also contained a wide range of compounds, especially chlorogenic acid, hesperidin, gallic acid, and ferulic acid. The major phytochemical in orange juice was hesperidin, but there were also substantial amounts of didymin and ferulic acid, whereas apple juice contained predominantly chlorogenic acid, along with phoredzin, catechin, and ferulic acid.

**Figure 1:**
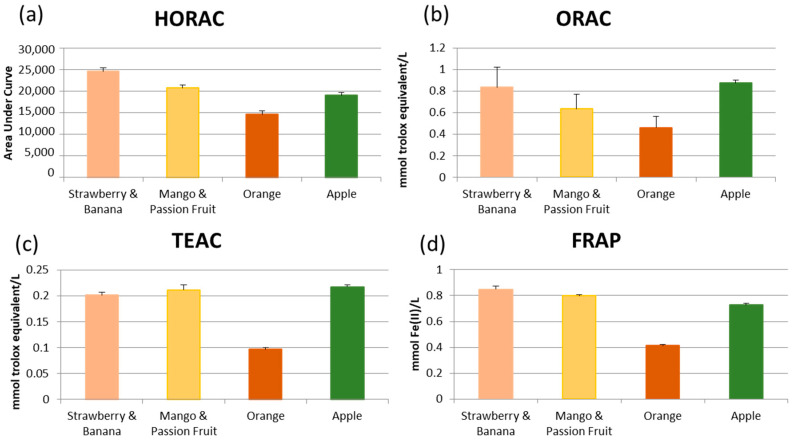
Antioxidant activity of juice/smoothie samples measured by four independent methods; ferric reducing ability of plasma (FRAP), the trolox equivalent antioxidant capacity (TEAC), oxygen radical absorbance capacity (ORAC) and the hydroxyl radical antioxidant capacity (HORAC). Data is presented as mean ± standard deviation (n = 3). All comparisons were significantly different (*p* < 0.05) with the exception of the ORAC data for strawberry and banana smoothie compared to apple juice.

To establish whether the differences in chemical composition had any impact on bioactivity a general screen of their antioxidant activity with four established methods was conducted (Figure 1). Redox activity a frequently attributed though still not necessarily a de facto mechanistically justified, wide benefit from the consumption of plant-based foods and the phytochemicals therein, including as an indicator of potential for participation in several biological pathways such as those involved in inflammation [11]. All comparisons of the data shown in Figure 1 were significantly different (*p* < 0.05) with the exception of the ORAC data for strawberry and banana smoothie compared to apple juice. Orange juice was consistently shown to have the lowest antioxidant activity across all assays, with apple juice, mango and passion fruit smoothie, and strawberry and banana smoothie significantly (*p* < 0.05) higher across all four methods. This is consistent with the availability of the phytochemicals studied, as in orange juice these are predominantly bound and only likely to be bioavailable after delivery and metabolism in the colon.

## 4. Discussion

Many of the phytochemicals measured in the smoothies and fruit juices have a demonstrated bioactivity, albeit mostly in vitro and in animal models. Hesperidin and didymin are flavonoid glycosides commonly found in citrus and other fruits, which have demonstrated antioxidant and potential anti-cancer tumourigenic activities in vitro [12,13,14,15,16]. It is interesting to note that hesperidin is mostly in the free form in the smoothies, whereas in the juice samples it is predominantly in the bound form. This suggests that the bioavailable hesperidin in the smoothies was likely liberated during production, whereas in the fruit juices, it remained bound to a soluble (or suspended) component during production. This is particularly important, as when bound to other components in the plant matrix, it is unlikely to be available for absorption early in the gastrointestinal (GI) tract where it can influence systemic health or impact on the gut transport of other important molecules. In orange juice, where this is a major metabolite, it may be that much of the hesperidin will be delivered to the colon, where it is likely to be extensively metabolized by the gut microbiota. This could have direct consequences for gut health, but also the metabolites once released and transformed could enter the systemic system through hepatic portal circulation.

Phloridzin is a dihydrochalcone glucoside, which has demonstrated blood glucose reduction in an animal model [17]. Phloridzin was found in substantial amounts in both smoothies and apple juice in the free form, where it would be potentially available to inhibit glucose uptake early in the GI tract. Chlorogenic acid is the quinic acid ester of caffeic acid, an antioxidant, which has been shown to have antihypertensive effects [18]. For all samples, chlorogenic acid was predominantly in the free form and as such, is likely to be absorbed early in the GI tract. As this is a major metabolite in apple juice it could have an impact on postprandial metabolism and health. Gallic acid is a trihydroxylated benzoic acid that has potent antioxidant activity [19]. Ferulic acid, p-coumaric acid, and caffeic acid are derivatives of cinnamic acid. Ferulic and caffeic acid exhibit antioxidant activity and all three compounds have shown anti-inflammatory activity in vitro.^19^ The hydroxycinnamic acids ferulic acid, p-coumaric acid, and caffeic acid were predominantly bound. These compounds are commonly found esterified to or cross-linked to plant cell wall polymers. The method of production whether as a juice or a smoothie did not impact availability and for both beverages, the majority of the hydroxycinnamic acids are most likely being delivered to the colon. Once metabolized by the gut microbiota and released, the metabolites produced have been shown to be anti-inflammatory at low concentrations [20]. Epicatechin and catechin are the flavan-3-ol epimers shown to have antioxidant activity and to confer protection from atherosclerotic lesion development and ischemic stroke damage in animal models [21,22]. Epicatechin reduces lipid peroxidation and inhibits platelet aggregation and blood vessel dilation by regulating nitric oxide [23]. The catechins were found to be highest in the strawberry and banana smoothie mostly in the free form, as was the case for the mango and passion fruit smoothie and the apple juice. This demonstrates availability in these beverages for a systemic effect. No catechins were detected in orange juice.

The study does not measure the antioxidant activity contribution of the individual compounds, but screens for overall antioxidant activity of the juices and smoothies. This demonstrates the potential for a bioactive impact, which is not only dependent on the composition of the beverages, but also reflect processing and the importance of the form in which the compounds are available from the matrix. This is an important consideration not only when planning interventions, but also when measuring the active components in food products.

Overall, fruit juices and smoothies were found to be rich in bioactive phytochemicals. In general, the smoothies contained a wider range of compounds whereas the juices were found to contain fewer compounds in the higher concentration range (>0.1 mg/mL). The fruit juices contained large amounts of single phytochemicals, namely hesperidin in orange juice and chrorogenic acid in apple juice. This is likely to reflect the fact that smoothies retain much more of the plant cell wall material, whereas the compounds measured in the juices were those solubilised or suspended during the juicing process. As there is much debate regarding the contribution of fruit juices and smoothies to the diet, an important consideration should be the potential wider benefits, including aspects of processing. However, short-term (acute) human dietary interventions are necessary to establish the bioavailability of these compounds to both the gut and systemic circulation, as well as postprandial markers such as those of satiety and glucose/insulin regulation. These interventions will inform longer-term (chronic) human studies that are essential to evaluate biomarkers of human health including the impact on the gut microbiota (an emerging critical determinant in the bioactivity of ingested phytochemicals reaching the large intestine) and longer-term markers of lipid metabolism, inflammation, and blood pressure. Interventions with fruit-based beverages should focus on specific mechanisms depending on the fruits from which they are comprised and most importantly the form in which the target components are available.

## Figures and Tables

**Table 1:** Benzoic acid derivative content of juice/smoothie samples.

	Strawberry and Banana		Mango and Passion Fruit		Orange		Apple	
	Free	Bound	Free	Bound	Free	Bound	Free	Bound
benzoic acid	12.46 ± 1.58	69.06 ± 7.87	14.20 ± 2.32	88.96 ± 19.83	12.18 ± 4.26	115.60 ± 20.20	11.62 ± 0.95	46.45 ± 13.93
salicylic acid	13.92 ± 1.58	46.08 ± 11.03	2.25 ± 0.53	12.76 ± 4.56	n/d	3.37 ± 0.96	0.14 ± 0.24	5.01 ± 1.97
4-hydroxy benzoic acid	42.15 ± 2.25	356.83 ± 30.46	7.37 ± 1.55	45.96 ± 3.95	2.41 ± 0.13	30.33 ± 4.00	1.31 ± 1.14	19.58 ± 2.27
2,3-dihydroxy benzoic acid	0.57 ± 0.04	13.56 ± 0.87	n/d	12.18 ± 2.16	n/d	5.81 ± 0.89	0.06 ± 0.10	7.45 ± 2.02
2,5-dihydroxy benzoic acid	3.22 ± 0.30	23.57 ± 2.16	0.88 ± 0.26	22.58 ± 1.29	n/d	7.97 ± 1.09	0.05 ± 0.09	18.96 ± 1.38
2,6-dihydroxy benzoic acid	3.38 ± 0.24	n/d	n/d	n/d	n/d	n/d	n/d	n/d
protocatechuic acid	34.56 ± 1.83	101.82 ± 3.24	12.85 ± 1.93	83.21 ± 9.22	1.39 ± 0.30	13.43 ± 1.70	4.74 ± 0.78	84.46 ± 10.44
gallic acid	127.29 ± 10.75	170.69 ± 17.33	687.02 ± 88.00	754.70 ± 90.55	0.23 ± 0.21	0.96 ± 0.14	1.86 ± 0.20	3.31 ± 0.72
vanillic acid	7.10 ± 0.32	48.77 ± 2.68	4.20 ± 1.03	40.22 ± 3.82	2.40 ± 0.33	45.29 ± 5.79	0.64 ± 0.07	14.97 ± 2.29
syringic acid	5.50 ± 0.32	73.46 ± 5.42	2.68 ± 0.27	27.96 ± 2.71	0.13 ± 0.23	3.43 ± 0.52	0.24 ± 0.03	11.12 ± 1.30
p-hydroxy benzaldehyde	4.19 ± 0.56	19.91 ± 2.37	1.48 ± 0.18	5.82 ± 0.87	1.68 ± 0.49	8.46 ± 1.73	3.54 ± 0.79	1.82 ± 0.44
protocatachaldehyde	2.90 ± 0.17	67.40 ± 5.57	3.00 ± 0.33	35.70 ± 8.40	0.44 ± 0.22	2.43 ± 0.28	9.04 ± 1.57	24.41 ± 4.02
3,4,5-trihydroxy benzaldehyde	n/d	105.71 ± 18.32	n/d	97.67 ± 25.72	n/d	n/d	n/d	n/d
vanillin	3.22 ± 0.54	8.98 ± 1.14	2.55 ± 0.40	10.20 ± 2.16	5.31 ± 0.66	13.75 ± 1.96	3.86 ± 0.71	1.97 ± 0.28
isovanillin	n/d	1.60 ± 0.63	n/d	1.50 ± 0.53	n/d	13.25 ± 3.41	n/d	n/d
syringin	2.61 ± 0.28	10.01 ± 1.98	2.07 ± 0.23	10.57 ± 1.55	3.06 ± 0.26	20.65 ± 3.97	0.48 ± 0.04	1.21 ± 0.10
2-hydroxy cinnamyl alcohol	n/d	0.51 ± 0.45	n/d	n/d	n/d	n/d	n/d	n/d

Data (µg/100 mL) is presented as mean ± standard deviation (n = 3). m-Hydroxybenzoic acid, 2,4-dihydroxybenzoic acid, 3,5-dihydroxybenzoic acid, o-anisic acid, m-anisic acid, p-anisic acid, 3,4-dimethoxybenzoic acid, 3-methoxybenzaldehyde, 3,4-dimethoxybenzaldehyde, and 3,4,5-trimethoxybenzaldehyde were not detected in any samples.

**Table 2:** Cinnamic acid derivative content of juice/smoothie samples.

	Strawberry and Banana		Mango and Passion Fruit		Orange		Apple	
	Free	Bound	Free	Bound	Free	Bound	Free	Bound
cinnamic acid	47.08 ± 3.54	83.36 ± 3.88	9.86 ± 0.91	26.67 ± 2.83	4.92 ± 3.00	14.74 ± 2.21	5.11 ± 2.95	13.86 ± 1.04
p-coumaric acid	128.31 ± 9.44	707.56 ± 54.01	17.34 ± 1.26	193.35 ± 30.85	8.72 ± 0.51	269.12 ± 54.50	6.87 ± 1.28	87.47 ± 7.76
caffeic acid	37.07 ± 2.95	124.98 ± 10.63	20.41 ± 3.14	243.78 ± 40.27	1.80 ± 0.18	44.65 ± 8.37	27.34 ± 2.66	297.41 ± 27.24
ferulic acid	12.78 ± 0.68	1096.74 ± 89.99	13.79 ±2.33	1012.24 ± 151.18	26.88 ± 2.20	528.87 ± 103.53	2.04 ± 0.40	56.92 ± 6.43
sinapic acid	16.24 ± 1.52	104.37 ± 4.83	13.60 ± 2.26	138.13 ± 22.32	31.78 ± 2.51	372.05 ± 77.99	0.17 ± 0.29	28.69 ± 3.53
3,4,5-trimethoxy cinnamic acid	0.39 ± 0.09	0.99 ± 0.12	0.12 ± 0.21	1.04 ± 0.28	0.38 ± 0.04	3.06 ± 0.73	n/d	n/d
4-hydroxy-3-methoxy cinnamyl alcohol	n/d	3.35 ± 3.71	n/d	3.03 ± 3.88	3.41 ± 2.04	2.77 ± 1.17	n/d	n/d
3-hydroxy phenylpropionic acid	n/d	0.99 ± 1.72	n/d	n/d	0.58 ± 1.00	n/d n/d	n/d	n/d
4-hydroxyphenyl propionic acid	n/d	15.42 ± 13.39	n/d n/d	17.21 ± 12.35	n/d n/d	9.76 ± 2.94	n/d	24.60 ± 7.42
4-hydroxy-3-methoxy phenylpropionic acid	0.58 ± 0.05	7.72 ± 2.19	n/d n/d	4.96 ± 0.25	0.73 ± 1.27	9.49 ± 1.28	n/d	1.40 ± 0.10
phenyllactic acid	4.68 ± 0.25	12.27 ± 0.72	5.95 ± 0.62	29.32 ± 19.31	10.60 ± 4.13	36.37 ± 4.07	4.03 ± 1.58	10.00 ± 1.27
4-hydroxy phenyllactic acid	11.55 ± 1.55	32.29 ± 2.69	14.62 ± 2.30	36.52 ± 3.30	6.31 ± 1.53	26.31 ± 1.60	4.85 ± 0.03	21.47 ± 4.65
ethylferulate	n/d	0.16 ± 0.27	n/d	n/d	0.07 ± 0.13	0.30 ± 0.09	n/d	n/d
coniferyl alcohol	8.32 ± 0.83	n/d n/d	0.51 ± 0.03	n/d	0.30 ± 0.30	n/d	n/d	n/d

Data (µg/100 mL) is presented as mean ± standard deviation (n = 3). o-Coumaric acid, m-coumaric acid, 3-methoxycinnamic acid, 4-methoxycinnamic acid, 3,4-dimethoxycinnamic acid, phenylpropionic acid, 2-hydroxyphenylpropionic acid, 3,4-dihydroxyphenylpropionic acid, and 3-methoxyphenylpropionic acid were not detected in any samples.

**Table 3:** Phenolic dimer, lignan, and acetophenone content of juice/smoothie samples.

	Strawberry and Banana		Mango and Passion Fruit		Orange		Apple	
	Free	Bound	Free	Bound	Free	Bound	Free	Bound
ferulic dimer (5-5 linked)	n/d	27.99 ± 1.38	n/d	17.53 ± 2.45	n/d	n/d	n/d	n/d
ferulic dimer (8-5 linked)	n/d	25.62 ± 1.80	n/d	13.90 ± 2.83	n/d	n/d	n/d	n/d
secoisolariciresinol	0.57 ± 0.12	0.15 ± 0.25	n/d	n/d	0.29 ± 0.19	n/d	n/d	n/d
matairesinol	0.16 ± 0.14	9.20 ± 0.75	n/d	10.68 ± 2.72	0.59 ± 0.57	3.60 ± 0.42	0.21 ± 0.11	5.67 ± 1.71
syringaresinol	13.18 ± 1.56	n/d	14.43 ± 2.61	n/d	3.60 ± 0.86	n/d	n/d	n/d
pinoresinol	0.87 ± 0.21	n/d	0.77 0.18	1.76 ± 1.63	0.66 ± 0.46	0.07 ± 0.12	0.58 ± 0.16	0.12 ± 0.10
lariciresinol	n/d	n/d	n/d	n/d	n/d	n/d	0.71 ± 0.72	n/d n/d
4-hydroxy acetophenone	0.80 ± 0.05	3.27 ± 0.18	n/d	1.71 ± 0.14	n/d	1.43 ± 0.10	0.02 ± 0.03	0.33 ± 0.03
4-hydroxy-3-methoxy acetophenone	0.33 ± 0.06	1.66 ± 0.09	n/d	1.25 ± 0.16	n/d	1.15 ± 0.07	n/d	0.24 ± 0.03
4-hydroxy-3,5-dimethoxy acetophenone	n/d	6.21 ± 0.48	n/d	6.02 ± 1.09	0.14 ± 0.01	3.76 ± 1.12	n/d	0.82 ± 0.11
3,4,5-trimethoxy acetophenone	0.04 ± 0.00	0.01 ± 0.01	0.08 ± 0.05	0.12 ±0.01	0.03 ± 0.01	n/d 0.01	n/d	0.02 ± 0.01

Data (µg/100 mL) is presented as mean ± standard deviation (n = 3). Ellagic acid, ferulic dimer (8-8 linked), resveratrol, hydroxymatairesinol, and 3,4-dimethoxyacetophenone were not detected in any samples.

**Table 4:** Phenylacetic acid, mandelic acid, and other phenolic metabolite content of juice/smoothie samples.

	Strawberry and Banana		Mango and Passion Fruit		Orange		Apple	
	Free	Bound	Free	Bound	Free	Bound	Free	Bound
phenylacetic acid	4.13 ± 0.36	10.51 ± 2.12	4.22 ± 0.06	13.77 ± 1.64	3.28 ± 1.03	11.20 ± 1.76	2.64 ± 0.92	9.85 ± 1.01
3-hydroxy phenylacetic acid	n/d	0.25 ± 0.43	n/d	n/d	0.20 ± 0.35	n/d	n/d	n/d
4-hydroxy phenylacetic acid	5.57 ± 1.26	32.69 ± 1.98	6.48 ± 0.89	51.05 ± 4.77	4.12 ± 0.96	41.36 ± 4.15	2.59 ± 0.35	22.18 ± 4.29
3,4-dihydroxy phenylacetic acid	n/d	9.86 ± 0.88	n/d	4.29 ± 0.99	n/d	n/d	n/d	4.77 ± 1.07
mandelic acid	n/d	n/d n/d	n/d	252.73 ± 15.15	n/d	n/d	n/d	n/d
3-hydroxy mandelic acid	n/d	116.81 ± 7.24	n/d	183.42 ± 12.71	n/d	166.90 ± 23.58	n/d	80.35 ± 14.61
4-hydroxy mandelic acid	37.51 ± 3.15	109.49 ± 13.98	n/d	121.38 ± 13.55	n/d	78.64 9.32	n/d	61.49 ± 7.86
3,4-dihydroxy mandelic acid	n/d n/d	7.96 ± 1.15	n/d	4.70 ± 0.56	n/d	n/d n/d	n/d	3.96 ± 0.43
anthranilic acid	0.73 ± 0.12	2.91 ± 0.39	n/d	0.60 ± 0.05	n/d	0.83 ± 0.11	n/d	0.37 ± 0.08
quinadilic acid	0.13 ± 0.05	0.52 ± 0.06	0.08 0.01	0.38 ± 0.04	0.10 ± 0.01	0.47 ± 0.12	0.03 0.01	0.20 ± 0.03
chlorogenic acid	621.95 ± 31.89	n/d n/d	1938.03 ± 236.28	1.07 ± 0.56	1.69 ± 0.21	0.20 ± 0.17	3107.85 ± 1029.43	4.42 ± 2.53
2-hydroxy hippuric acid	n/d	0.06 ± 0.10	n/d	n/d	0.04 ± 0.06	n/d	n/d	n/d
p-cresol	n/d	2.05 ± 0.64	n/d	6.20 ± 1.34	n/d n/d	1.97 ± 0.68	n/d n/d	4.92 ± 1.19
4-ethylphenol	0.28 ± 0.04	2.67 ± 0.92	0.18 0.17	2.85 ± 0.27	0.10 ± 0.09	1.48 ± 0.52	0.10 0.09	1.92 ± 0.27
4-methyl catechol	0.06 ± 0.01	6.28 ± 0.69	0.11 0.04	10.55 ± 2.84	0.09 ± 0.02	4.92 ± 1.48	0.13 ± 0.02	6.69 ± 1.10
hydroxy tyrosol	5.88 ± 0.45	15.89 ± 13.38	1.06 0.14	6.04 ± 2.31	n/d n/d	0.74 ± 0.18	0.09 ± 0.01	0.56 ± 0.39
tyrosol	5.36 ± 1.00	32.94 ± 1.70	n/d	18.35 ± 1.99	1.59 ± 0.08	20.11 ± 3.84	n/d	5.41 ± 0.63

Data (µg/100 mL) is presented as mean ± standard deviation (n = 3). 4-Hydroxy-3-methoxyphenylacetic acid, 4-methoxyphenylacetic acid, and 4-hydroxy-3-methoxymandelic acid were not detected in any samples.

**Table 5:** Indole and polyamine content of juice/smoothie samples.

	Strawberry and Banana		Mango and Passion Fruit		Orange		Apple	
	Free	Bound	Free	Bound	Free	Bound	Free	Bound
	1.47 ± 0.06	0.13 ± 0.23	0.30 ± 0.51	n/d	n/d	n/d n/d	0.19 ± 0.21	n/d
indole-3-acetic acid	n/d	0.13 ± 0.02	n/d	0.16 ± 0.04 ±	n/d	0.09 ± 0.01	n/d	0.05 ± 0.01
indole-3-acrylic acid	0.65 ± 0.10	n/d	0.42 ± 0.08	n/d	n/d	n/d n/d	n/d	n/d
indoe-3-lactic acid	n/d	53.12 ± 6.61	n/d	42.90 ± 6.53 ±	n/d	n/d n/d	n/d	n/d
pyrollidine	n/d	n/d	n/d	n/d	n/d	5.20 ± 0.56	n/d	n/d
piperidine	1.47 ± 0.06	0.13 ± 0.23	0.30 ± 0.51	n/d	n/d	n/d n/d	0.19 ± 0.21	n/d

Data (µg/100 mL) is presented as mean ± standard deviation (n = 3). Indole, indole-3-propionic acid, indole-3-carbinol, indole-3-carboxylic acid, indole-3-methyl, spermine, spermidine, tyromine, histamine, cadaverine, putresine, and 5-hydroxytryptophan were not detected in any samples.

**Table 6:** Psoralen, coumarin, coumesterol, isoflavone, and catechin content of juice/smoothie samples.

	Strawberry and Banana		Mango and Passion Fruit		Orange		Apple	
	Free	Bound	Free	Bound	Free	Bound	Free	Bound
8-methylpsoralen	n/d	n/d	1.90 ± 0.16	n/d	n/d	n/d	n/d	n/d
bergapten	12.78 ± 2.37	n/d	55.36 ± 4.82	0.75 ± 0.06	n/d	n/d	n/d	n/d
coumarin	n/d	14.08 ± 7.16	n/d	22.33 ± 2.54	n/d	17.47 ± 3.30	n/d	24.30 ± 2.58
umbelliferone	n/d	3.75 ± 0.25	n/d	2.75 ± 0.08	0.33 ± 0.02	37.06 ± 3.34	n/d	n/d
scopoletin	n/d	1.74 ± 0.14	n/d	2.44 ± 0.06	0.20 ± 0.01	11.32 ± 0.73	n/d	n/d
phloretin	0.37 ± 0.06	n/d	0.35 ± 0.04	n/d	n/d	n/d	0.53 ± 0.04	0.08 ± 0.04
imperatorin	0.08 ± 0.01	0.08 ± 0.01	0.36 ± 0.17	0.15 ± 0.03	0.01 ± 0.01	n/d	n/d	0.07 ± 0.04
phloridzin	146.89 ± 10.51	1.16 ± 0.25	258.11 ± 36.99	3.14 ± 0.84	0.13 ± 0.02	n/d	396.65 ± 26.88	4.61 ± 0.02
formononetin	1.13 ± 0.56	10.96 ± 8.75	0.54 ± 0.12	26.62 ± 26.62	0.43 ± 0.29	4.95 ± 1.67	0.57 0.24	9.17 ± 7.19
daidzein	n/d	n/d	n/d	0.31 ± 0.04	n/d	n/d	n/d	0.28 ± 0.06
biochanin A	0.05 ± 0.00	0.07 ± 0.02	0.03 ± 0.01	0.03 ± 0.01	0.03 ± 0.01	0.04 ± 0.04	0.03 ± 0.01	0.01 ± 0.01
catechin	259.08 ± 16.87	3.66 ± 4.20	85.03 ± 16.02	7.46 ± 6.49	n/d	n/d	93.54 ± 4.93	23.22 ± 6.46
epicatechin	108.28 ± 7.89	n/d	284.70 ± 57.59	3.53 ± 3.08	n/d	n/d	380.75 ± 19.53	18.19 ± 4.54
gallocatechin	5.46 ± 0.38	4.75 ± 0.47	n/d	5.01 ± 0.45	n/d	2.34 ± 0.33	n/d n/d	3.33 ± 0.34

Data (µg/100 mL) is presented as mean ± standard deviation (n = 3). 4-Methylumbelliferone, psoralen, 7-hydroxy-4-methylcoumarin, 4-hydroxy-6-methylcoumarin, 7,8-dihydroxy-6-methylcoumarin, coumesterol, isoliquiritigenin, genstein, epigallocatechin, and epigallocatechin gallate were not detected in any samples.

**Table 7:** Flavonoid content of juice/smoothie samples.

	Strawberry and Banana		Mango and Passion Fruit		Orange		Apple	
	Free	Bound	Free	Bound	Free	Bound	Free	Bound
eriocitrin	14.17 ± 0.69	0.52 ± 0.16	14.46 ± 2.78	1.06 ± 0.25	94.05 ± 6.78	10.62 ± 3.97	n/d	n/d
naringenin	0.73 ± 0.04	2.66 ± 0.72	0.71 ± 0.07	3.51 ± 0.61	0.87 ± 0.05	30.69 ± 16.22	0.09 ± 0.01	n/d
hesperitin	0.83 ± 0.06	9.51 ± 1.02	0.64 ± 0.05	11.01 ± 1.38	0.31 ± 0.01	98.43 ± 47.51	0.09 ± 0.02	0.17 ± 0.02
kaempferol	4.14 ± 0.28	31.62 ± 6.04	n/d	n/d	n/d	5.05 ± 1.23	n/d	n/d
morin	0.44 ± 0.76	n/d n/d	n/d n/d	10.45 ± 0.82	n/d	96.58 ± 46.35	n/d	n/d
quercetin	9.82 ± 4.51	11.19 ± 1.61	3.20 ± 0.46	4.57 ± 0.64	n/d	26.98 ± 8.54	1.73 ± 0.16	0.39 ± 0.11
myricetin	0.89 ± 0.13	0.16 ± 0.28	n/d	n/d	n/d	n/d n/d	n/d n/d	n/d n/d
quercetin-3-glucoside	41.51 ± 3.41	2.49 ± 0.22	47.87 ± 7.93	3.07 ± 0.54	1.97 ± 0.09	9.40 ± 1.42	59.52 ± 3.58	1.71 ± 0.09
taxifolin	1.57 ± 0.06	1.84 ± 0.22	0.54 ± 0.06	1.19 ± 0.18	0.05 ± 0.09	4.79 ± 1.03	0.07 ± 0.06	0.45 ± 0.22
hesperidin	1210.11 ± 70.13	287.76 ± 76.76	1069.10 ± 143.52	473.28 ± 39.57	1720.84 ± 48.63	1967.66 ± 394.88	2.02 ± 0.15	4.42 ± 0.17
quercitrin	31.91 ± 2.63	0.24 ± 0.04	51.65 ± 7.45	0.38 ± 0.08	n/d	n/d	108.91 ± 8.90	0.44 ± 0.07
didymin	140.50 ± 9.41	9.28 ± 2.85	128.77 ± 17.64	17.23 ± 2.18	390.46 ± 7.93	183.11 ± 64.69	0.39 ± 0.04	0.09 ± 0.02
luteolin	n/d	n/d	n/d	n/d	n/d	2.17 ± 0.38	n/d	n/d
luteolinidin	63.03 ± 7.92	17.44 ± 1.36	19.14 ± 2.91	19.60 ± 1.71	n/d	9.88 ± 0.60	30.17 ± 2.49	19.49 ± 2.79
isorhamnetin	n/d	7.18 ± 0.36	n/d	5.89 ± 0.51	n/d	54.20 ± 14.67	n/d	n/d
apigenin	0.35 ± 0.03	1.23 ± 0.09	0.03 ± 0.02	0.95 ± 0.02	0.01 0.02	8.07 ± 1.39	0.04 ± 0.00	n/d
gossipin	2.90 ± 0.20	0.91 ± 0.24	n/d n/d	n/d n/d	0.56 0.19	1.20 ± 0.27	1.03 ± 0.14	n/d
tangeretin	89.50 ± 16.29	3.22 ± 2.04	112.12 ± 13.08	1.56 ± 0.32	310.53 ± 17.34	11.92 ± 2.70	n/d	n/d
cyanidin-3-galactoside	12.81 ± 1.39	23.71 ± 4.23	7.56 ± 0.77	27.13 ± 4.43	9.83 ± 0.10	61.94 ± 2.84	2.73 ± 0.16	15.21 ± 5.35
peonidin-3-glucoside	5.53 ± 0.68	4.26 ± 1.78	n/d	3.51 ± 0.66	n/d	4.39 ± 0.99	n/d	4.39 ±1.16
petunidin-3-glucoside	7.78 ± 0.51	9.99 ± 0.14	5.23 ± 0.75	12.83 ± 1.22	7.56 ± 0.32	23.18 ± 1.36	10.72 ± 0.16	19.40 ± 1.95

Data (µg/100 mL) is presented as mean ± standard deviation (n = 3). Neohesperidin, naringin, galangin, fisetin, neoeriocitrin, poncirin, and peonidin were not detected in any samples.

## Supplementary Materials

The following are available online at https://www.mdpi.com/2304-8158/9/7/891/s1, Figure S1: Representative chromatograms of flavonoids detected by negative ion MS for each of the fruit beverages; A. Strawberry and banana Smoothie, B. Mango and Passion Fruit Smoothie, C. Orange Juice and D. Apple Juice; Table S1: Transition Values; Declustering Potential (DP), Collision energy (CE), Collision cell exit Potential (CXP) and Retention Times (t_R_) for all analytes.
