# Supplementary material for: Fruit-Based Beverages Contain a Wide Range of Phytochemicals and Intervention Targets Should Account for the Individual Compounds Present and Their Availability
Source: Foods. 2020 Jul 7;9(7):891. doi: 10.3390/foods9070891 (PMC7404635; doi:10.3390/foods9070891)
Supplement: Supplementary file 1 [file foods-09-00891-s001.pdf]

A.

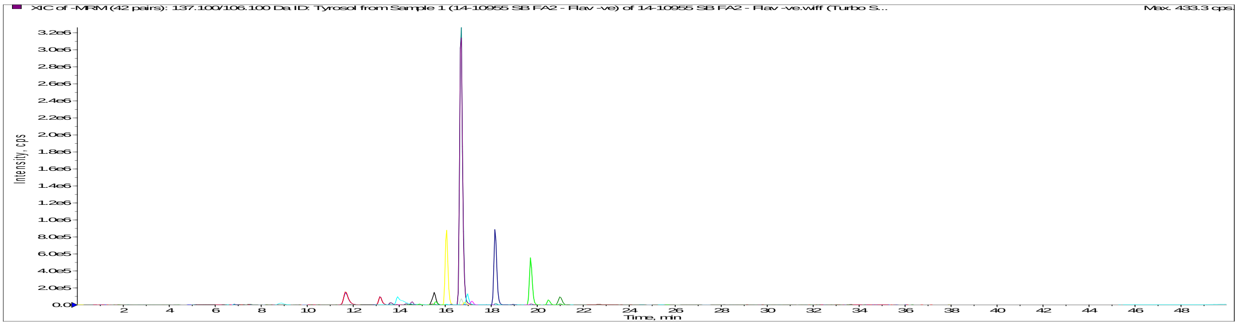

B.

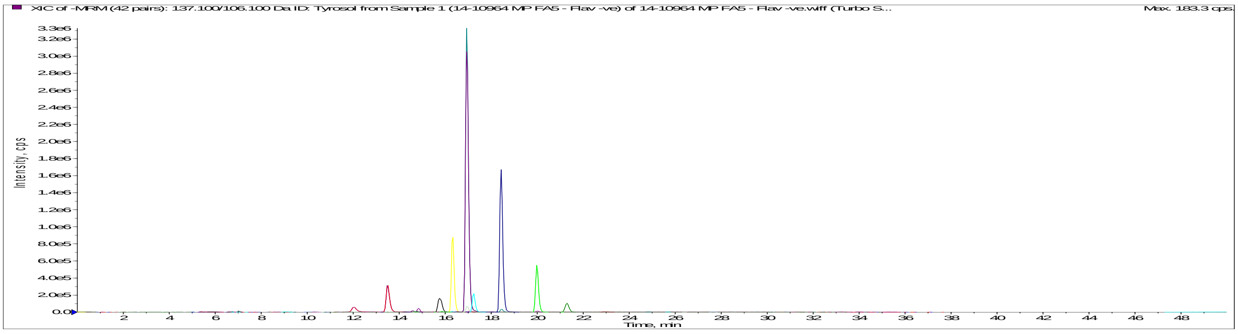

C.

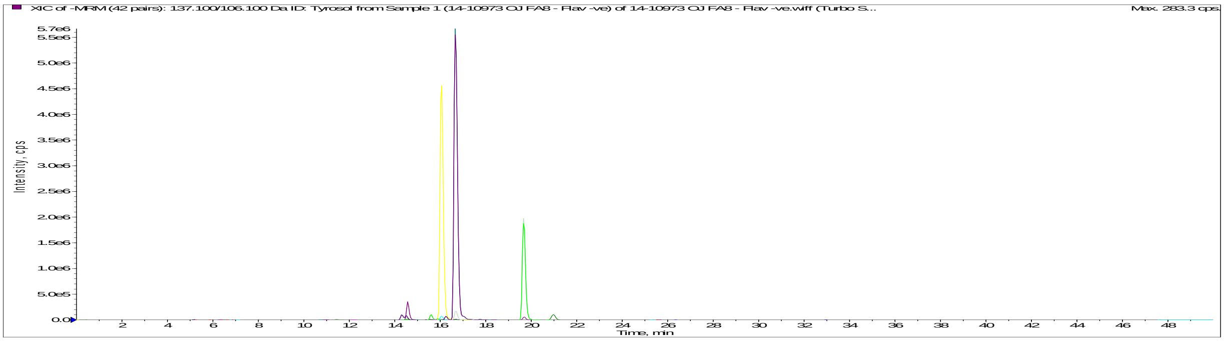

D.

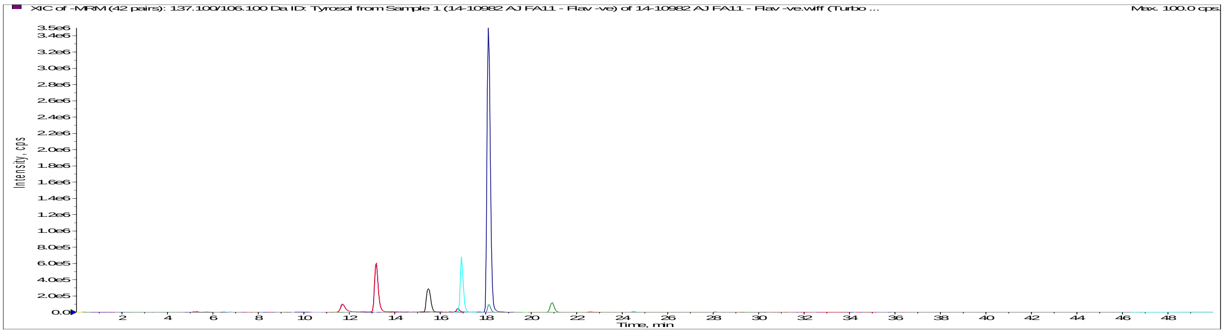

Supplementary Figure 1: Representative chromatograms of flavonoids detected by negative ion MS for each of the fruit beverages; A. Strawberry and banana Smoothie, B. Mango and Passion Fruit Smoothie, C. Orange Juice and D. Apple Juice.

Supplementary Table 1: Transition Values; Declustering Potential (DP), Collision energy (CE), Collision cell exit Potential (CXP) and Retention Times (t_R) for all analytes.

	Q1 Mass	Q3 Mass	DP	CE	CXP	t_R	Method
**benzoic acid**	121.2	77.2	-40	-16	-4.8	9.62	1
**salicylic acid**	137.1	93.1	-30	-24	-1.72	29.64	2
**m-hydroxybenzoic acid**	137.1	93.1	-30	-24	-1.72	20.85	2
**p-hydroxybenzoic acid**	137.1	93.1	-30	-24	-1.72	17.71	2
**2,3-dihydroxybenzoic acid**	153.1	109.1	-32.5	-21	-1.2	17.81	2
**2,4-dihydroxybenzoic acid**	153.1	109.1	-32.5	-21	-1.2	18.97	2
**2,5-dihydroxybenzoic acid**	153.1	109.1	-32.5	-21	-1.2	15.45	2
**2,6-dihydroxybenzoic acid**	153.1	109.1	-32.5	-21	-1.2	22.86	2
**protocatechuic acid**	153.1	109.1	-32.5	-21	-1.2	12.78	2
**3,5-dihydroxybenzoic acid**	153.1	109.1	-32.5	-21	-1.2	11.68	2
**o-anisic acid**	151.1	107.1	-36	-17	-2.4	27.06	2
**m-anisic acid**	151.1	107.1	-36	-17	-2.4	33.38	2
**p-anisic acid**	151.1	107.1	-36	-17	-2.4	32.3	2
**gallic acid**	169.1	125.1	-36	-20.3	-1.2	6.83	2
**vanillic acid**	167.1	152.1	-41	-23	-3.6	19.17	2
**syringic acid**	197.1	182.1	-30	-19	-2.7	19.19	2
**3,4-dimethoxybenzoic acid**	181.1	137.1	-35.5	-17.5	-1.3	26.68	2
**p-hydroxybenzaldehyde**	121.1	92.1	-48	-35	-2.7	22.9	2
**protocatachaldehyde**	137.1	108.1	-56	-32	-2.4	17.28	2
**3,4,5-trihydroxybenzaldehyde**	153.1	124.1	-50.5	-31	-1.26	10.9	2
**vanillin**	151.1	136.1	-31	-18	-3	24.62	2
**isovanillin**	151.1	136.1	-31	-18	-3	24.06	2
**syringin**	181.1	166.1	-31.5	-17	-2.18	24.99	2
**3-methoxybenzaldehyde**	137.1	109.1	54	15	4.4	14.67	4
**3,4-dimethoxybenzaldehyde**	167.1	123.7	56	26	3.5	12.04	4
**3,4,5-trimethoxybenzaldehyde**	197.1	137.6	53	22	4.9	13.29	4
**cinnamic acid**	147.1	103.1	-34.5	-15.65	-1.9	38.15	2
**o-coumaric acid**	163.1	119.1	-31	-22.5	-2.7	30.09	2
**m-coumaric acid**	163.1	119.1	-31	-22.5	-2.7	27.18	2
**p-coumaric acid**	163.1	119.1	-31	-22.5	-2.7	24.09	2
**caffeic acid**	179.1	135.1	-35.52	-23.48	-2.19	19.15	2
**ferulic acid**	193.1	134.1	-40	-23	-3.3	25.57	2
**sinapic acid**	223.1	164.1	-42	-22	-2.2	24.99	2
**3-methoxycinnamic acid**	177.1	103.1	-43	-18	-2.1	39.69	2
**4-methoxycinnamic acid**	177.1	117.1	-36	-41.2	-1.5	38.33	2
**3,4-dimethoxycinnamic acid**	207.1	103.1	-36	-20	-2.4	32.71	2
**3,4,5-trimethoxycinnamic acid**	237.1	103.1	-48	-22	-2.6	35.16	2

**phenylpropionic acid**	149.1	105.1	-38	-15.5	-7.85	37.66	2
**2-hydroxyphenylpropionic acid**	165.1	121.1	-38	-16	-9	28.54	2
**3-hydroxyphenylpropionic acid**	165.1	121.1	-38	-16	-9	25.38	2
**4-hydroxyphenylpropionic acid**	165.1	121.1	-38	-16	-9	22.93	2
**3,4-dihydroxyphenylpropionic acid**	181.1	137.1	-44	-16.5	-1.6	18.02	2
**4-hydroxy-3-methoxyphenylpropionic acid**	195.1	136.1	-47	-23	-2.4	24.24	2
**3-methoxyphenylpropionic acid**	179.1	119.1	-42	-18	-8.4	37.81	2
**phenol**	93.1	65.1	-49	-28.5	-9.4	5.22	3
**1,2-hydroxybenzene**	109.1	91.1	-55	-28	-2.1	18.93	2
**1,3-hydroxybenzene**	109.1	65.1	-27	-18	-2.6	15.41	2
**1,2,3-trihydroxybenzene**	125.1	79.1	-60	-26	-1.5	10.14	2
**4-hydroxyacetophenone**	135.1	92.1	-48.5	-32.5	-1.53	24.43	2
**4-hydroxy-3-methoxyacetophenone**	165.1	150.1	-35.5	-20	-1.9	26.13	2
**4-hydroxy-3,5-dimethoxyacetophenone**	195.1	180.1	-32	-20	-2.1	26.12	2
**3,4-dimethoxyacetophenone**	181.1	123.6	54	27	5.7	12.18	4
**3,4,5-trimethoxyacetophenone**	211.1	154.1	47	24	7.2	13.31	4
**phenylacetic acid**	135.1	91.1	-19	-10	-0.6	31.39	2
**3-hydroxyphenylacetic acid**	151.1	107.1	-21.5	-13.5	-1.17	20.86	2
**4-hydroxyphenylacetic acid**	151.1	107.1	-21.5	-13.5	-1.17	18.6	2
**3,4-dihydroxyphenylacetic acid**	167.1	123.1	-17.5	-13	-1.5	13.9	2
**4-hydroxy-3-methoxyphenylacetic acid**	181.1	137.1	-25	-10.5	-1.3	19.91	2
**4-methoxyphenylacetic acid**	165.1	106.1	-21	-18.9	-1	32.12	2
**mandelic acid**	151.1	107.1	-31	-15	-2.7	17.09	2
**3-hydroxymandelic acid**	167.1	121.1	-32	-31.5	-9.2	9.41	2
**4-hydroxymandelic acid**	167.1	121.1	-32	-31.5	-9.2	6.59	2
**3,4-dihydroxymandelic acid**	183.1	137.1	-40	-21	-1.5	5.8	2
**4-hydroxy-3-methoxymandelic acid**	197.1	137.1	-30	-27	-2.4	7.33	2
**phenylpyruvic acid**	165.1	147.2	-48	-16	-6.3	6.88	1
**4-hydroxyphenylpyruvic acid**	179.1	107.1	-35	-17	-5	2.4	3
**phenyllactic acid**	165.1	147.2	-48	-16	-6.3	7.27	1
**4-hydroxyphenyllactic acid**	181.1	135.1	-39.5	-23	-9.4	13.16	2
**anthranilic acid**	138.1	64.5	45	36	5.1	10.42	4
**quinadilic acid**	174.2	128.1	50	31	11.4	6.96	4
**chlorogenic acid**	353.2	191.1	-37.9	-29	-2.5	14.5	2
**0-hydroxyhippuric acid**	194.1	93.1	-32	-33	-2	23.89	2
**ethylferulate**	221.1	206.1	-43	-23	-3	45.66	2
**3OMe4OHBAlc**	153.1	135.1	-31	-15.5	-5.5	3.35	3

**p-cresol**	107.1	77.1	-57	-27.5	-4.6	6.22	3
**4-ethylphenol**	121.1	106.1	-61	-20	-7.6	7.62	3
**4-methylcatechol**	123.1	108.1	-49	-25.5	-6.4	4.52	3
**tyrosol**	137.1	106.1	-29	-23	-2.3	12.27	6
**hydroxytyrosol**	153.1	123.1	-38	-21	-2.8	8.75	6
**ellagic acid**	301.2	145.1	-111	-53	-9.8	8.19	1
**ferulic dimer (5-5 linked)**	385.1	282.1	-58	-31	-5.8	7.43	1
**ferulic dimer (8-8 linked)**	385.1	282.1	-58	-31	-5.8	11.26	1
**ferulic dimer (8-5 linked)**	385.1	282.1	-58	-31	-5.8	6.49	1
**ferulic dimer (5-5 hydrogenated)**	389.1	329.2	-126	-40	-6.6	7.4	1
**reservatrol**	227.1	143.1	-57	-36	-3.2	21.15	6
**indole**	118.1	91.1	80	32	3.1	11.03	4
**indole-3-acetic acid**	174.1	130.1	-27.62	-16.49	-1.4	31.77	2
**indole-3-acrylic acid**	188.2	170.1	51	19	9.6	37.04	4
**indole-3-propionic acid**	188.2	59	-54.5	-23	-0.5	11.62	2
**indole-3-carbinol**	146.1	128.1	-22	-16.5	-5	4.11	3
**indole-3-carboxylic acid**	160.1	116.1	-31.6	-25	-1	28.73	2
**indole-3-pyruvic acid**	202.1	130.1	-49	-14.5	-4.6	2.43	3
**indole-3-methyl**	132.1	116.7	77	29	4.8	16.94	4
**indole-3-lactic acid**	206.1	117.7	62	31	4.8	9.73	4
**5-hydroxytryptophan**	177.1	160.1	21	15.7	2.7	6.57	7
**coumarin**	147.1	91.1	49.5	34	1.6	16.1	5
**psoralen**	187.1	131.1	54	31.5	2.4	17.94	5
**8-methylpsoralen**	217.2	202.1	54	28	3.6	18.23	5
**bergapten**	217.2	202.101	54	28	3.6	19.41	5
**tangeretin**	373.1	343.3	60	35	5.5	21.71	5
**coumesterol**	269.1	213.1	73	35	4	16.27	5
**catechin**	289.2	109.1	-54	-35	-1.8	11.53	6
**epicatechin**	289.2	109.1	-54	-35	-1.8	13	6
**gallocatechin**	305.1	125.1	-49	-30	-3	7.41	6
**epigallocatechin**	305.1	125.1	-49	-30	-3	8.83	6
**epigallocatechin gallate**	457.1	169.1	-36	-25.5	-2.4	13.62	6
**isoliquiritigenin**	255.1	135.1	-40	-23	-3.8	27.01	6
**phloretin**	273.1	167.1	-43	-23	-4.2	24.36	6
**imperatorin**	271.1	203.1	59	14	5.3	18.55	6
**eriocitrin**	595.4	151.1	-62	-53	-1.8	14.38	6
**naringenin**	271.1	151.1	-52	-26.5	-2.2	25.16	6
**naringin**	579.3	271.1	-91	-44.5	-3.2	16.34	6
**hesperitin**	301.1	164.1	-56	-33	-1.8	16.52	6
**kaempferol**	285.1	93.1	-85	-49	-2.2	25.45	6
**morin**	301.1	125	-56	-29.5	-2.8	25.86	6
**quercetin**	301.1	179.1	-51	-27	-1.8	22.51	6

**myricetin**	317.1	151.1	-75	-35	-3.2	19.48	6
**quercetin-3-glucoside**	463.1	300.1	-93	-41	-4.2	15.36	6
**taxifolin**	303.1	125.1	-52	-31.5	-2.8	17.78	6
**genstein**	269.1	133.1	-69	-43.5	-1.4	25.1	6
**scopoletin**	191.1	176.1	-26	-21.5	-2	17.7	6
**umbelliferone**	161.1	133.1	-51	-29	-1.4	18.24	6
**7,8-dihydroxy-6-methyl coumarin**	207.1	192.1	-42	-22	-2.2	14.55	6
**neohesperidin**	609.5	301.1	-91	-47	-4.4	16.94	6
**hesperidin**	609.5	301.1	-91	-47	-4.4	25.86	6
**quercitrin**	447.1	300.1	-71	-37	-4.4	16.79	6
**biochanin A**	283.1	268.1	-59	-31	-4	32.39	6
**poncirin**	593.3	285.1	-99	-51	-3.2	19.97	6
**didymin**	593.3	285.1	-99	-51	-3.2	19.57	6
**peonidin**	301.2	286.2	70.9	31.7	5.06	5.99	5
**phloridzin**	435.1	167.1	-46	-43	-2.4	17.99	6
**daidzein**	253.1	91.1	-72	-51	-1.8	21.26	6
**galangin**	269.1	169.1	-69	-37	-2.6	32.15	6
**luteolin**	285.1	133.1	-86	-45.5	-3.4	22.01	6
**equol**	241.1	121.1	-44	-20	-2.8	25.34	6
**fisetin**	285.1	135.1	-60	-28.5	-2.8	19.56	5
**luteolinidin**	271.1	115.1	82	67	1.8	6.78	6
**neoeriocitrin**	595.3	151.1	-99	-53	-4	14.82	6
**isorhamnetin**	315.1	300.1	-68	-31.5	-4.2	25.71	6
**formononetin**	267.1	252.1	-57	-30.5	-4.8	27.94	6
**apigenin**	269.1	117.1	-61	-52.5	-2.8	24.66	6
**gossypin**	479.1	317.1	-59	-31.5	-3.8	17.97	6
**glycitein**	285.1	270.2	70	35	4.5	13.82	5
**secoisolariciresinol**	361.1	165.1	-88	-34.5	-7.1	6.89	1
**matairesinol**	357.1	83.1	-72	-38	-5.1	11.3	1
**enterodiol**	301.1	253.1	-80	-31	-4.4	8.15	1
**enterolactone**	297.1	107.1	-86	-39	-3.3	11.8	1
**syringaresinol**	417.1	181.1	-65	-28	-8.4	8.96	1
**pinoresinol**	357.1	151.1	-72	-25	-6.5	10.04	1
**lariciresinol**	359.1	160.1	-41	-40	-7	7.7	1
**hydroxymatairesinol**	373.1	173.1	-65	-41	-8	7.76	1
